# Mapping Chinese Medical Entities to the Unified Medical Language System

**DOI:** 10.34133/hds.0011

**Published:** 2023-03-30

**Authors:** Luming Chen, Yifan Qi, Aiping Wu, Lizong Deng, Taijiao Jiang

**Affiliations:** ^1^ Guangzhou Laboratory, Guangzhou, China.; ^2^ Guangzhou Medical University, Guangzhou, China.; ^3^ Institute of Systems Medicine, Chinese Academy of Medical Sciences & Peking Union Medical College, Beijing, China.; ^4^ Suzhou Institute of Systems Medicine, Suzhou, China.

## Abstract

**Background:**

Chinese medical entities have not been organized comprehensively due to the lack of well-developed terminology systems, which poses a challenge to processing Chinese medical texts for fine-grained medical knowledge representation. To unify Chinese medical terminologies, mapping Chinese medical entities to their English counterparts in the Unified Medical Language System (UMLS) is an efficient solution. However, their mappings have not been investigated sufficiently in former research. In this study, we explore strategies for mapping Chinese medical entities to the UMLS and systematically evaluate the mapping performance.

**Methods:**

First, Chinese medical entities are translated to English using multiple web-based translation engines. Then, 3 mapping strategies are investigated: (a) string-based, (b) semantic-based, and (c) string and semantic similarity combined. In addition, cross-lingual pretrained language models are applied to map Chinese medical entities to UMLS concepts without translation. All of these strategies are evaluated on the ICD10-CN, Chinese Human Phenotype Ontology (CHPO), and RealWorld datasets.

**Results:**

The linear combination method based on the SapBERT and term frequency-inverse document frequency bag-of-words models perform the best on all evaluation datasets, with 91.85%, 82.44%, and 78.43% of the top 5 accuracies on the ICD10-CN, CHPO, and RealWorld datasets, respectively.

**Conclusions:**

In our study, we explore strategies for mapping Chinese medical entities to the UMLS and identify a satisfactory linear combination method. Our investigation will facilitate Chinese medical entity normalization and inspire research that focuses on Chinese medical ontology development.

## Introduction

Well-developed medical terminology systems, such as the Unified Medical Language System (UMLS), are the cornerstone of medical informatics research and health informatics technology for facilitating fine-grained medical knowledge representation and other high-level intelligent applications for medicines [1–3]. A large number of unorganized Chinese medical terms remain challenges for Chinese medical informatics development and applications [4]. Therefore, it is necessary to build a high-quality unified terminology system to facilitate Chinese medical information processing for research and for clinical improvement.

The UMLS, which was developed by the National Institutes of Health, is a set of files and software that aggregates more than 200 health and biomedical vocabularies and standards to enable interoperability between systems [5]. With the aid of lexical analysis tools [6], terms with the same meaning from different vocabularies are manually linked as a concept by medical professionals [7]. However, most medical terms (70.8%) in the UMLS are in English. Only 10% of medical terms in UMLS are in Spanish. The ratio of French medical terms in the UMLS is approximately 2.7%, and only 485 Hebrew medical terms are collected in the UMLS [8]. The simplified Chinese version LOINC is the only Chinese vocabulary that has been included in the UMLS. For most non-English speaking countries, there are no resources, such as time, finance, and human expertise, for constructing unified terminology systems from scratch, as the National Institutes of Health has done. Consequently, there is a demand of countries lacking the integration between medical vocabularies to develop computational methods to accomplish the unification of medical terminologies. Previous studies have shown that computational methods for mapping cross-lingual medical entities to the UMLS are effective. Perez-Miguel et al. [9] matched Spanish medical terms to the UMLS through lexical transformation. Spanish medical entities recognized from electronic health records were normalized as the UMLS concepts. Bitton et al. [8] transliterated the UMLS terms into a variety of candidate Hebrew sequences using a transliteration model. Then, medical entities extracted from online Hebrew health communities were linked to the UMLS concepts based on Hebrew transliterations. However, Chinese is a type of logographic writing system and is different from Latin languages (such as English, Spanish, and French). None of the above methods are applicable for mapping Chinese medical entities to the UMLS. The most intuitive solution for mapping Chinese medical entities to the UMLS is the translation method. Ruan et al. [10] translated Chinese medical entities into English by calling the Baidu Fanyi. Then, the Jaccard distance (a metric measuring the similarity between 2 strings) between translations and the UMLS concepts was calculated for mapping. However, the translation quality is not robust due to the diversity of Chinese medical entities. It is difficult to map Chinese medical entities to the UMLS comprehensively by only comparing the Jaccard distance. In Ruan’s study, 4,298 (26,821 in total) Chinese medical entities were mapped to the UMLS [10]. The result shows that the translation method comparing the Jaccard distance is not a sufficient way to map Chinese medical entities to the UMLS.

With the development of deep learning, pretrained language models (PLMs), such as BERT [11], GPT [12], and T5 [13], have been widely used in various fields of natural language processing (NLP). In medical informatics research, PLMs trained on a large medical corpus are usually used to represent medical terms at the semantic level. Medical-domain-specific PLMs not only learn the character features of medical terms but also acquire semantic information related to the context of medical terms. For example, the LexLM model presented by Nguyen et al. [14] achieved state of the art (SOTA) on the UMLS Vocabulary Alignment task by representing the UMLS concepts for a high-dimensional embedding space (BioWordVec [15]). In addition, due to the PLM’s ability to perform transfer learning, it has been shown that knowledge, such as synonymous and hierarchical relationships, defined in the knowledge graph can be infused into PLMs training with a specific contrastive learning task [16]. Liu et al. [17] presented a contrastive metrics learning method to learn the self-alignment of UMLS concepts and trained a language model called SapBERT. SapBERT achieved SOTA in many biomedical entity-linking tasks. PLMs representing medical terms into embedding space overcome the difficulty of mapping medical terms with substantial differences at the character level as a concept, which facilitates medical terminology normalization and interoperability.

The methods discussed above have proven to be effective in medical informatics applications, such as the normalization of English medical terms. There is still a gap in the exploration of the effective solution for mapping Chinese medical entities to the UMLS. Hence, in this study, we investigate the technical boundaries of mapping Chinese medical entities to the UMLS by systematically exploring string-based, semantic-based, and string-semantic combination mapping strategies. In the following sections, we first detail the data collection, evaluation metrics, and implementation of techniques used in this study. Then, we analyze the mapping results of different strategies. Finally, we propose a potentially satisfactory solution for mapping Chinese medical entities to the UMLS. In addition, the solution we propose is not only applicable for Chinese medical entities, but it can also be inspiring and helpful for countries facing similar challenges. For more details about implementation and evaluation, please visit https://github.com/Yifan-haddock/CMCN. Although we only implement and test mapping strategies on medical datasets in simplified Chinese, all methods we discuss in this study can be applied to a wide range of non-English languages, including traditional Chinese, Korean, Japanese, and Arabic.

## Methods

**Fig. 1.**
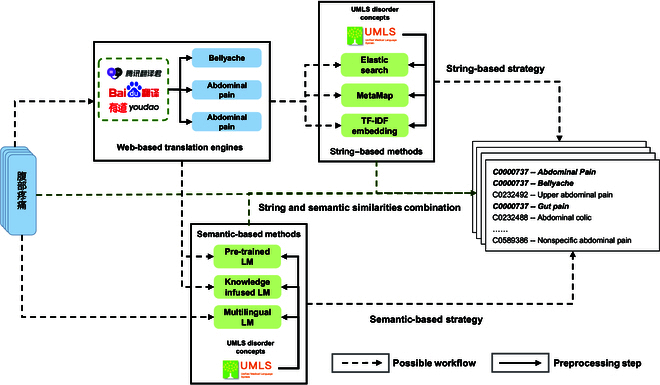
All possible strategies for mapping Chinese medical entities to the UMLS. String-based strategy: We translate original Chinese medical entities to English first and map translated queries to the UMLS by applying MetaMap [18], Elasticsearch (ES), and the term frequency-inverse document frequency (TF-IDF) [19] bag-of-words (TF-IDF BoW) model [20]. Candidate concepts are selected according to the mapping scores. Semantic-based strategy: We employ translated queries as input to obtain semantic similarities between queries and UMLS concepts and rank candidate concepts according to the semantic similarity scores. Additionally, without translation, we employ original Chinese queries as input and compute semantic similarities between original queries and UMLS concepts using cross-lingual PLM. Candidate concepts are ranked and selected according to similarity scores. Integration strategy of string and semantic similarity: We compute string and semantic similarities between translated queries and UMLS concepts simultaneously, combine similarity scores as the final scores by using a variety of integration schemes and rank candidate concepts accordingly.

Figure 1 briefly describes all mapping strategies we investigate in this study. Chinese medical entities were taken as original queries. Concepts defined as “Disorders” in UMLS formed a candidate concepts dictionary.

### Evaluation data and metrics

To evaluate the mapping performance of different strategies, 3 datasets are applied.

1. ICD10-CN. The ICD-10 simplified Chinese version was created by the National Health Committee (NHC) in China and contains 11,451 disease terms. All of these medical terms were included in the ICD10-CN dataset. Terms in the dataset were mapped to the UMLS through the ICD10-AM codex defined in the UMLS Metathesauruses [7].

2. CHPO. The CHPO ontology was created by the CHPO organization, which focuses on the development and management of the Chinese Human Phenotype Ontology (CHPO). All 13,655 terms in the CHPO thesaurus were collected into the evaluation dataset and mapped to the UMLS according to the Human Phenotype Ontology [21] codex in the UMLS.

3. The RealWorld dataset. To evaluate the mapping performance of strategies in real-world applications (medical entities that were used in clinical electronic health records, online health communities, case reports, or other documents in real world usage), we manually constructed an evaluation dataset called the RealWorld dataset that contains 2,824 medical terms. All medical terms in the dataset were collected from real-world medical documents. Y.Q. and L.C. manually mapped these medical terms to the UMLS. L.D. reviewed the mapping results. Details about the construction and quality assessment of the RealWorld dataset are discussed in the supplementary materials (Section S2 Evaluation Dataset) and annotation guideline.

Additionally, the mapping performance of every strategy was assessed by using the top *n* accuracy noted as *Acc@n* [22]. In this manuscript, we use *Acc@1*, *Acc@5*, and *Acc@10* to quantify the performance. The calculation of *Acc@n* is described as follows:

$$\mathrm{Acc}@n=\frac{{\mathrm{TP}}_{n}}{N}∙100$$ (1)

where *n* denotes the top *n* candidate concepts that are selected to compare with true CUI labels. *N* denotes the number of terms in the evaluation dataset. *TP_n_* denotes the number of terms that are correctly mapped to the UMLS concepts according to the top-*n* recommendations.

### Multiple-source translations

Benefiting from the development of deep learning NLP techniques, the performance of web-based translation engines is rapidly increasing [23–25]. In this study, we introduce a multiple source (including Baidu Fanyi, Youdao, and Tencent Translator) translation scheme as a key step for mapping Chinese medical entities to the UMLS. Translation qualities are evaluated on a high-quality biomedical Chinese–English translation dataset (32,554 medical terms) released by NHC. All medical terms in this dataset are translated and reviewed by medical professionals. The quality evaluation of translations is discussed in the supplementary materials (Section S1). Additionally, we observed that the translations that do not match standard English terms could also explain the real meaning of the Chinese medical term. For example, “脂沉积症” means disturbance of lipid metabolism with abnormal deposits of lipids in the cells. The translation result of this Chinese medical term is “Lipid Deposition Disorder” and “Liposis”. The standard translation in the NHC dataset is “lipid storage disease”. Although the spelling of these translations varies, they both describe the same disease concept. Thus, translation variants produced by multiple translation engines facilitate mapping Chinese medical entities to UMLS concepts.

### String-based strategy

Since the full development of medical terminology systems in English, such as UMLS, string-based methods have become the primary solutions for entity recognition and normalization in English [26]. MetaMap is a well-known NLP tool for biomedical document annotation. ES is a kind of search engine based on the Lucene BM25 algorithm. MetaMap and ES are commonly used in entity-linking tasks in English [27–29]. In this study, we apply MetaMap and ES for mapping Chinese medical entities to the UMLS with the aid of translations. In addition, we also utilize a TF-IDF BoW model fitted on UMLS to map entities to UMLS concepts. Differences between these string-based methods are fully discussed in the supplementary materials (Section S3).

#### MetaMap method

Supported by vast UMLS Metathesauruses, MetaMap implements word disambiguation, lexical lookup, and variant identification algorithms, which enables MetaMap to recognize medical entities from free-text documents and link them to the UMLS concepts. Furthermore, with the “-Z” parameter (short segmentation normalization), MetaMap can be applied for entity normalization tasks. In this study, we use MetaMap with the “-Z” parameter and limit the semantic type of concepts to “disorders” with the “-J” option. The top *n* (*n* = {1, 5, 10}) concepts recommended by MetaMap are taken as the mapping results. The principle and implementation details of the MetaMap method in this study are listed in the supplementary materials (Section S3.1).

#### ES method

ES is a search engine implementation based on Lucene. The core searching algorithm of ES is Okapi BM25, a string-based algorithm for entity matching. In our study, we extract all disorder concepts from the “MRCONSO” table of UMLS2020AB and transform all concepts into the ES index. To map Chinese medical entities, all entities are translated to English queries to search candidate concepts through the ES index using the fuzzy matching model. The top *n* (*n* = {1, 5, 10}) recommended concepts are taken as mapping results. We discuss the main principle of the Okapi BM25 algorithm and implementation details of ES in the supplementary materials (Section S3.2).

#### TF-IDF BoW method

The TF-IDF BoW model is commonly used to represent tokens and their contextual information as vectors through a matrix of subword fragments from a large corpus. The TF-IDF of subword fragments can be calculated as follows:

$$\mathrm{tf}\left(\mathrm{st},d\right)=\frac{f_{\mathrm{st},d}}{\sum_{t\prime \in d}f_{t\prime ,d}}$$ (2)

where *f*_*st*, *d*_ is the raw count of an *n*-gram in a document, and *tf*(*st*, *d*) represents the frequency of the term.

$$\mathrm{idf}\left(\mathrm{st},D\right)=\log\frac{N}{\left|\left\{d\in D:\mathrm{st}\in d\right\}\right|}$$ (3)

*N* is the total number of documents in the corpus. |{*d* ∈ *D* : *st* ∈ *d*}| represents the number of documents where the term *st* appears. *idf*(*st*, *D*) represents the inverse document frequency of the term in the corpus. Finally, TF-IDF can be calculated as follows:

tfidf(st,d,D)=tf(st,d)·idf(st,D) (4)

We fit the *n*-gram TF-IDF BoW model to the UMLS disorder vocabularies using the scikit-learn [30] Python package. Parameter “*n*” for *n*-grams is set to 2 and 3 in the training process. Translated medical entities and UMLS concepts are represented as high-dimensional sparse vectors using TF-IDF BoW models. Where the vector length equals the *n*-gram dictionary length. Each entry in a sparse vector equals an *n*-gram TF-IDF value in the corpus if that *n*-gram occurs in the term being transformed or 0 if it does not. Then, the similarity scores between translated queries and candidate concepts are calculated by using the cosine similarity:

$$S_{\text{string}}(q,c)=\cos\left(\theta \right)=\frac{e_{q}^{s}\cdot e_{c}^{s}\;}{|e_{q}^{s}\;\|e_{c}^{s}\;|}\;$$ (5)

where *e^s^* represents sparse vectors of query and candidate concepts. Finally, the top *n* (*n* = {1, 5, 10}) UMLS candidates are selected as the mapping recommendations. Details about the TF-IDF BoW model are illustrated in the supplementary materials (Section S3.3).

### Semantic-based strategy

Semantic word embeddings or PLMs trained with a large corpus represent the word and its context information in high-dimensional vector space [31]. Words closer to each other in this type of vector space are expected to have similar meanings [31]. Many methods can be used for obtaining word embeddings, such as Word2Vec [31], GloVe [32], and BERT [11]. BERT-based methods, developed in recent years, have been proven to outperform multiple NLP tasks [33]. Lee et al. [34] and Gu et al. [35] found that language models fine-tuned with PubMed documents achieve better performance in many domain-specific NLP tasks. Moreover, knowledge-infused language models have been developed in recent years, benefiting from the establishment of contrastive metric learning schemes. Liu et al. trained a language model called SapBERT [17] with whole UMLS knowledge using a pairwise self-alignment training method. SapBERT achieved SOTA on many entity-linking benchmarks. Therefore, methods assessing the semantic similarity between Chinese medical entities (or their translations) and UMLS concepts can be applied for the mappings. In this study, we include BERT, BioBERT [34], and SapBERT language models to accomplish the mapping of Chinese medical entities to the UMLS. All queries and UMLS candidate concepts are encoded as follows:

$$e^{d_{\mathrm{LM}}}=\mathrm{LM}\left(\overset{⃐}{t}\right)\left[\mathrm{CLS}\right]$$ (6)

We apply cosine similarity to quantify the semantic similarity between queries and candidates (Eq. 7) and ranked concepts accordingly. Then, the top *n* (*n* = {1, 5, 10}) UMLS concepts are taken as the recommendation mappings for Chinese medical entities.

$$S_{\text{semantic}}\left(q,c\right)=\cos\left(\theta \right)=\frac{e_{q}^{d}\cdot e_{c}^{d}\;}{|e_{q}^{d}\;\|e_{c}^{d}\;|}$$ (7)

where *e^d^* represents the semantic vectors of the query and the candidate encoded by the language model. *LM* means the language model. *CLS* is a special token generated by the language model, which usually stands for the last layer output of the language model for representing the meaning of the entire input sequence.

Additionally, multilingual-BERT (mBERT) [11] trained on cross-lingual corpora, such as wiki-data [36], has been proven to perform considerable improvements for many cross-lingual NLP tasks, such as cross-lingual entity linking, disambiguation, and knowledge alignment. Therefore, we also investigate the performance of cross-lingual semantic strategies for mapping Chinese medical entities to the UMLS without translations. Original Chinese queries and UMLS concepts are represented as vectors at the semantic level based on cross-lingual PLMs. Cosine similarity is also applied to measure the similarities between queries and UMLS concepts. In this study, we include mBERT, xlm-RoBERTa [37], and xlm-SapBERT [38] as cross-lingual language models to perform the mapping of Chinese medical entities to the UMLS without translations.

### String and semantic similarity integration strategies

As Ning et al. discussed in their study [39], there is a variety of combination methods to integrate string and semantic similarities. Thus, we designed 4 different integration methods—the z-score, min-max, tanh, and linear combination—to integrate string and semantic similarity scores, which consider both string and semantic contributions to the similarity between queries and candidate concepts. To compare with other mapping strategies, the performance of the integration strategy is also evaluated on the ICD10-CN, CHPO, and RealWorld datasets. The top *n* (*n* = {1, 5, 10}) candidate concepts were recommended according to the integrated similarity scores. MetaMap and ES were not tested in the integration strategy since their scoring mechanism is different from embedding-based cosine similarity scoring methods, such as the TF-IDF BoW method and semantic-based strategy. We only applied the best semantic method SapBERT and the best string method TF-IDF BoW as the integration component.

We implement z score, min-max, tanh, and linear combination methods separately in this study. All methods except the linear combination were implemented utilizing the scikit-learn framework. Following the marginal distribution optimization method described by Sung et al. in BioSYN [40], we implement the linear combination method with the PyTorch framework and train it on UMLS disorder datasets to optimize the combination parameters. The similarity scores between the query terms and candidate terms are calculated as follows:

$$S_{\text{integrate}}=\alpha S_{\text{semantic}}+\beta S_{\text{string}}$$ (8)

**Fig. 2.**
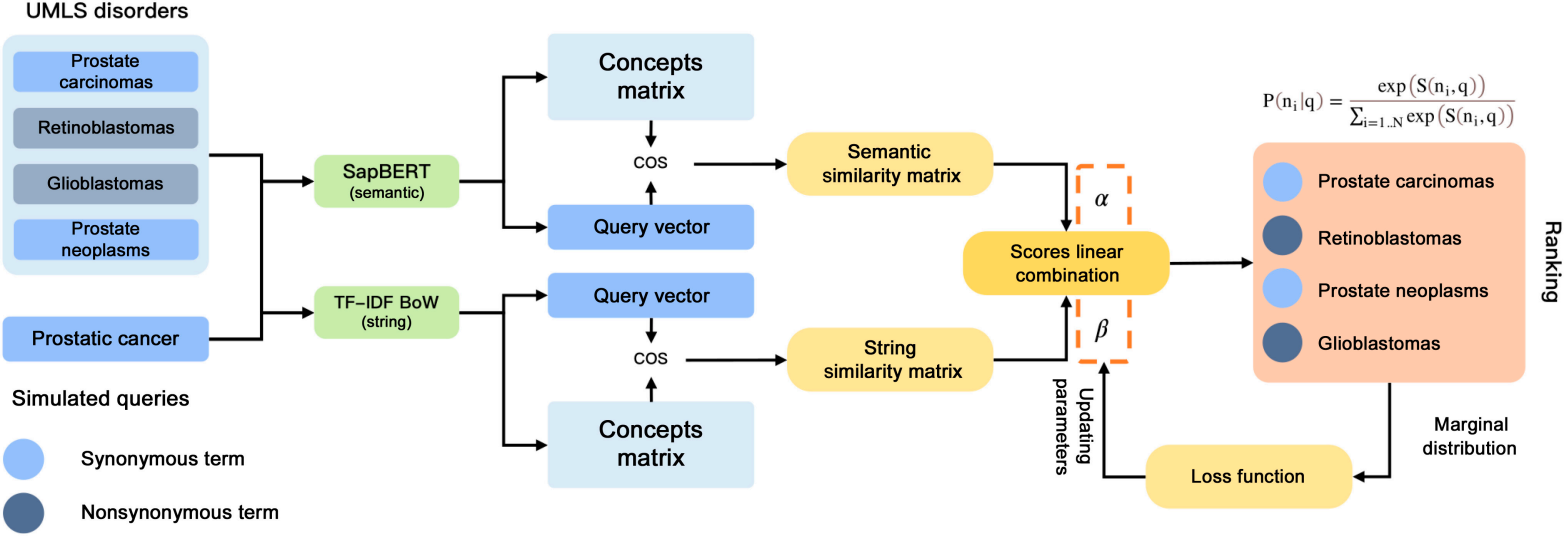
Overview of the training process for optimizing linear combination parameters.

Thus, optimizing the *α* and *β* parameters is essential to achieve the best performance. The training process for the optimization is briefly described in Fig. 2. Details about the linear combination method are described in the supplementary materials (Section S6).

First, we randomly select 10K entities from the disorder dataset and translate them to Chinese as a simulated dataset. Then, in the training process, the top 20 recommendations are selected from the disorder dictionary according to the integration of string and semantic similarities. After that, we calculate the marginal probability of positive synonymous in recommendations as follows:

$$P\left(n_{i},q_{s}\right)=\frac{\exp\left(S_{\text{integrate}}\left(n_{i},q_{s}\right)\right)}{\sum_{i=1...N\;}\exp\left(S_{\text{integrate}}\left(n_{i},q_{s}\right)\right)}$$ (9)

The marginal probability of positive synonymous terms in the recommendation set is defined as follows:

$$P^{\prime }\left(q_{s},N\right)=\sum_{n\in N;\text{EQUAL}\left(q_{s},n\right)=1}P\left(n_{i}|q_{s}\right)$$ (10)

where *EQUAL*(*q_s_*, *n*) = 1 means entities in the candidate set that are the synonymous terms to the target entity. Our goal is maximizing the marginal probability of positive synonymous terms in the recommendation set. Thus, the loss function is defined as follows:

$$\text{Loss}=-\frac{1}{Q}\sum_{i=1}^{Q}\log P^{\prime }\left(q_{s_{i}},N_{q_{s_{i}}}\right)$$ (11)

where *Q* is the number of entities in the training set. When the model was optimized, integrated similarity scores became greater for synonymous candidates and smaller for nonsynonymous candidates. The parameters, *α* and *β*, trained from the disorder simulation dataset finally converge at 33.11 and 7.28, respectively.

## Results

Overall, as shown in Table, the multiple unified translation is the critical point that promotes the performance for all strategies. The linear combination strategy with TF-IDF BoW and SapBERT models performs the best in our experiment. All strategies and their evaluation results are discussed in detail in the following sections.

**Table.** Strategies performance of mapping Chinese medical entities to the UMLS on ICD10-CN, CHPO, and the RealWorld datasets. Numbers with bold font illustrate the best performance in one method. We found that all methods with the aid of multiple-source translation perform best. Numbers with italic font style demonstrate the best performance in one strategy. The underlined numbers represent the optimal strategy among all we explored. McNemar’s test [41] was performed to statistically test significance of mapping performance difference between methods. More details about significant test are illustrated in the supplementary materials (Section S5). With the aid of multisource translation, linear-combination method based on SapBERT and TF-IDF BoW significantly outperformed other methods on both 3 evaluation datasets (numbers labeled with ** represent significant results).

Dataset and metrics		ICD10-CN			CHPO			RealWorld		
		Acc@1	Acc@5	Acc@10	Acc@1	Acc@5	Acc@10	Acc@1	Acc@5	Acc@10
Strategies	Translator									
String-based										
MetaMap	Baidu Fanyi	20	23	23.13	30.98	34.57	34.81	42.49	50	50.57
	Youdao	16.33	20.02	21.07	24.68	28.06	28.35	37.04	44.23	45.08
	Tencent Translator	19.47	22.59	22.79	29.31	32.97	33.25	40.72	48.34	49.11
	Multiple unified translation	**21.13**	**24.99**	**25.94**	**34.09**	**39.56**	**41.01**	**47.84**	**55.91**	**57.58**
Elasticsearch	Baidu Fanyi	50.88	66.05	71.78	42.14	55.04	59.12	52.51	62.71	65.97
	Youdao	44.32	60.05	66.33	36.49	49.69	54.1	46.18	56.02	59.67
	Tencent Translator	49.09	64.3	69.99	40.51	53.76	57.86	50.39	60.38	63.74
	Multiple unified translation	**54.23**	**65.92**	**71.6**	**46.82**	**59.31**	**63.95**	**55.17**	**66.61**	**70.08**
*n*-gram TF-IDF BoW (*n* = 2)	Baidu Fanyi	64.32	79.65	83.63	50.5	66.03	70.69	54.89	68.45	71.57
	Youdao	58.44	75.63	79.89	46.47	61.2	65.78	49.04	62.54	66.22
	Tencent Translator	63.51	78.58	82.34	49.65	65.14	69.79	52.55	66.64	69.97
	Multiple unified translation	**69.1**	**81.92**	**85.71**	**55.21**	**69.19**	**73.73**	**59.21**	**71.53**	**75.07**
*n*-gram TF-IDF Bow (*n* = 3)	Baidu Fanyi	64.22	80.83	85.08	49.82	65.99	70.87	54.5	68.94	72.24
	Youdao	59.78	77.9	82.15	45.38	61.78	66.82	48.62	63.46	67.14
	Tencent Translator	63.62	79.91	84.01	48.74	65.44	70.01	52.55	67.17	71.03
	Multiple unified translation	***68.56***	***82.61***	***86.47***	***54.47***	***69.22***	***74.23***	***58***	***72.49***	***75.6***
Semantic based										
BERT	Baidu Fanyi	43.58	55.5	59.35	34.01	43.24	46.28	48.09	57.4	59.17
	Youdao	35.62	47.2	51.42	26.37	34.51	37.5	39.7	48.26	50.21
	Tencent Translator	41.89	53.93	57.94	32.77	42.92	46.47	45.79	54.71	56.83
	Multiple unified translation	**49.17**	**59.15**	**62.99**	**39.84**	**49.9**	**52.87**	**53.29**	**63.17**	**65.16**
BioBERT	Baidu Fanyi	52.79	66.96	71.74	39.62	52.36	57.1	52.55	64.98	68.52
	Youdao	45.12	60.45	65.59	31.52	43.4	47.8	45.08	56.66	60.45
	Tencent Translator	51.12	66.2	70.84	38.33	51.6	56.56	50.35	62.61	65.97
	Multiple unified translation	**57.14**	**70.16**	**74.44**	**45.24**	**57.58**	**62.11**	**56.87**	**68.98**	**72.24**
SapBERT	Baidu Fanyi	72.4	87.59	90.51	58.53	74.53	78.9	59.53	74.68	77.83
	Youdao	69.96	85.25	88.47	54.43	70.72	75.85	53.97	68.91	72.38
	Tencent Translator	71.33	86.33	89.36	58.16	74.13	78.57	57.58	73.55	76.98
	Multiple unified translation	***75.51***	***88.35***	***91.01***	***63.39***	***77.08***	***81.36***	***63.92***	***77.62***	***81.69***
mBERT	None	0.05	0.11	0.22	0.08	0.24	0.34	0	0.04	0.07
xlm-RoBERTa	None	0.88	2.11	2.99	0.22	0.6	0.93	0.14	0.74	1.31
xlm-SapBERT	None	62.73	80.11	84.96	39.19	58.74	66.37	38.28	54.28	61.4
Integration strategies(SapBERT + TF-IDF BoW)										
z score	Baidu Fanyi	74.52	89.21	91.88	57.93	75.47	80.29	58.43	72.77	76.45
	Youdao	71.63	86.94	90.12	54.05	72.31	77.33	53.58	68.13	72.1
	Tencent Translator	74.66	88.7	91.23	57.76	75.21	80.21	56.62	71.81	75.74
	Multiple unified translation	**77.64**	**89.57**	**92.48**	**62.57**	**77.57**	**82.01**	**61.97**	**75.6**	**79.32**
Min-Max	Baidu Fanyi	75.46	89.68	92.26	59.61	77.12	81.67	59.84	74.15	77.97
	Youdao	72.85	87.48	90.67	56	74.05	79.09	54.14	69.62	73.55
	Tencent Translator	75.55	89.16	91.69	59.17	76.74	81.67	58.43	73.09	77.2
	Multiple unified translation	**78.55**	**90.1**	**92.73**	**64.28**	**78.5**	**83.09**	**63.17**	**76.7**	**80.38**
Tanh	Baidu Fanyi	74.57	89.22	91.88	57.94	75.53	80.29	58.6	72.8	76.59
	Youdao	71.68	86.94	90.12	54.15	72.32	77.36	52.66	68.2	72.2
	Tencent Translator	74.56	88.7	91.22	57.63	75.19	80.23	56.76	71.81	75.71
	Multiple unified translation	**77.7**	**89.59**	**92.49**	**62.76**	**77.58**	**82**	**62.22**	**75.53**	**79.43**
linear-combination	Baidu Fanyi	79.79	91.53	93.75	65.15	81.62	85.91	62.04	76.38	79.71
	Youdao	76.82	89.31	92.02	62.18	79.17	83.73	56.69	71.39	75.5
	Tencent Translator	79.52	91.24	93.32	65.19	81.82	86.05	60.66	75.14	79.14
	Multiple unified translation	81.86**	91.85**	94.1**	68.95**	82.44**	86.76**	64.77**	78.43**	81.91**

### String-based strategy

As shown in Table, the TF-IDF BoW method achieves better performance than MetaMap and ES and obtained Acc@5 accuracies of 82.61%, 69.22%, and 72.49% on the ICD10-CN, CHPO, and RealWorld datasets, respectively. TF-IDF BoW not only considers the character information of strings but also pays attention to the TF-IDF weights of *n*-gram subwords. For example, “肝内管梗阻” means the impairment of bile flow from the liver to the small intestine due to blockage of the biliary duct system; the translation results provided by translation engines are “intrahepatic duct obstruction” and “obstruction of the intrahepatic duct”. MetaMap and ES could not link this term to the correct CUI due to the complexity of medical terms. However, TF-IDF BoW methods could link “肝内管梗阻” to the correct CUI, “C0860211-Intrahepatic biliary obstruction” because important subwords, such as “-int-”, “-hep-”, and “-obs-” were weighted more in the TF-IDF BoW model. Furthermore, we also compare the performance of TF-IDF BoW models using different *n*-gram parameters (*n* = 2 or *n* = 3). As shown in Table, the TF-IDF BoW model with *n* = 3 outperforms the model with *n* = 2. However, because of memory limitation, we do not evaluate models with n > 3.

### Semantic-based strategy

Table also shows the evaluation for semantic methods. Compared with string-based methods, SapBERT outperforms in mapping Chinese medical entities to the UMLS. Acc@5 accuracy in ICD10-CN is 91.14%, in CHPO it is 82.06%, and in the RealWorld dataset it is 78.05%. SapBERT was enhanced by infusing synonymous knowledge from UMLS and inherited context information from PLMs focusing on the biomedical domain, such as PubMedBERT [35]. Thus, the SapBERT language model learned semantic information and synonymous knowledge at the same time. For example, “双肾盂” (translated to Double renal pelvis), which was mapped to the UMLS by SapBERT but failed by string-based methods. The standard concept of “双肾盂” is “duplication of renal pelvis”. The context information of “Double” and “Duplication” is relatively close in the corpus. However, string-based methods cannot capture the similarity features between them through character information and consequently performs worse than SapBERT. However, Table shows that the Acc@5 accuracy for BioBERT is 70.16% in ICD10-CN, 57.58% in CHPO, and 68.98% in the RealWorld dataset, and the BERT model achieved even worse results. Both BERT and BioBERT, which were only fine-tuned on biomedical corpora without infusing UMLS knowledge, perform worse than the TF-IDF BoW (*n* = 3) model. The TF-IDF BoW model was trained on the UMLS disorder dictionary, but the BERT and BioBERT language models were not. Thus, TF-IDF represents the features of the disorder dictionary much better than BERT and BioBERT, which promotes the mapping performance.

Additionally, we also evaluate the performance of cross-lingual language models. Chinese medical entities can barely be mapped to the UMLS without any translations by using general cross-lingual language models, such as mBERT and xlm-RoBERTa, as shown in Table. However, xlm-SapBERT trained with the UMLS full dataset showed comparable performance to other strategies. The Acc@5 accuracy in ICD10-CN is 80.11%, in CHOP it is 58.74%, and in the RealWorld dataset it is 54.28%. Due to the cross-lingual synonymous knowledge included in the UMLS full dataset, xlm-SapBERT pulls those medical terms with the same meanings from different languages into closer vector space. The xlm-SapBERT mapping strategy makes it possible to map cross-lingual medical entities to the UMLS without translations.

### String and semantic similarity integration strategy

In the above sections, we discuss string-based strategy and semantic-based strategy. However, string-based methods or semantic-based methods cannot correctly normalize medical terms alone in some situations. For example, in a semantic vector space, “type ii endometrial carcinoma” and “endometrial carcinoma stage ii” are both close to “endometrial cancer type ii”, which is translated from “子宫内膜癌 2 型”. If we only use the semantic-based methods, “endometrial carcinoma stage ii” will be mistakenly ranked as Top 1 in the recommendation concept due to the semantic and sequential similarity (BERT-like language models learn positional information from the corpus and become sensitive to the sequential order [42]), but the true concept is “type ii endometrial carcinoma”. The integration of string-based and semantic-based methods can solve this problem by considering the contributions of string and semantic similarities simultaneously.

As shown in Table, the linear combination method performs best, with 91.85%, 82.44%, and 78.43% Acc@5 accuracy in the ICD10-CN, CHPO, and RealWorld datasets, respectively, which is better than that of SapBERT. However, not all integration schemes provide performance increments for mapping entities to the UMLS. Other schemes, including the z score, min-max, and tanh methods, reduce the mapping ability and introduce some noise into the similarity scores. Based on the evaluation results, we can observe that the linear combination method performs best in mapping Chinese medical entities to the UMLS among all methods we investigate in this study.

In conclusion, the optimal method for mapping Chinese medical entities to the UMLS is the linear combination method with the aid of multiple-source web translation engines. In the linear combination method, the TF-IDF BoW (*n* = 3) model is applied for string information representation, and the SapBERT (knowledge-infused language model) model represents the semantic information. This method considers both string and semantic similarities and tuned optimal parameters to maximize the differences between positive and negative concept recommendations. Moreover, we surprisingly discovered that the knowledge-infused cross-lingual language model, xlm-SapBERT, achieves comparable performance in mapping tasks for Chinese medical entities and achieves a marvelous improvement compared with mBERT and xlm-RoBERTa. This discovery demonstrates that the cross-lingual strategy is helpful for cross-lingual medical entity linking when web-based translation engines cannot be reached, such as some isolated network environments in hospitals, due to security reasons. Our exploration investigates possible strategies for mapping Chinese medical entities to the UMLS, and we present a satisfactory choice under current technical support.

## Discussion

In this study, we focus on the exploration of potential effective strategies for mapping Chinese medical entities to the UMLS. According to the evaluation results on 3 datasets, the optimal method for the mapping is the linear combination method that integrates string (TF-IDF) and semantic (SapBERT) similarity scores between terms’ translations and UMLS concepts. The linear combination method achieves 91.85% Acc@5 on the ICD10-CN dataset, 82.44% Acc@5 on the CHPO dataset, and 78.43% Acc@5 on the RealWorld dataset.

It was found that the embedding-based approaches considerably outperform traditional string-based methods (MetaMap and ES). In contrast to the traditional methods that only focus on string similarity at the word level, embedding approaches represent the subword information and the contextual information where each token is located and learn the semantic features during the training process. Our experimental results demonstrate that semantic-based methods perform better in handling the case of medical entities with semantically similar but different spelling. For example, for the medical entities influenza (C0021400) and flu, although their strings are completely different, the contextual information they are in is close because the medical meanings they express are the same. The PLMs that learned contextual information by training with a large corpus can map influenza and flu into a similar semantic space. Furthermore, it was also found that the improvement brought by the semantic method with knowledge infusion (SapBERT) is obvious. SapBERT migrates different terms of the same concept to a closer distance in semantic vector space by setting the goal to learn synonymy relationships among UMLS terms. Terms with the same meaning form a packed cluster in the SapBERT embedding space. Moreover, the TF-IDF BoW (*n*-gram, *n* = 3) model that was trained on the UMLS also achieves comparable performance to some semantic methods, such as BioBERT, which further corroborates that the models’ performance on mapping tasks can be enhanced by learning UMLS knowledge. Therefore, in future research, considering fully utilizing the knowledge in the UMLS or other high-quality medical ontologies will provide opportunities to enhance the performance of PLMs on medical-entity standardization and cross-lingual medical-entity mapping tasks.

In addition, the most critical component of a biomedical ontology is the definition of relationships (including synonyms, hypernyms, hyponyms, modifications, interactions, etc.) between medical entities [43]. Our mapping approach can facilitate Chinese medical entities to inherit the relationships defined in the UMLS, such as CUI mappings, hierarchical relationships, concept definitions, and other relevant relationships between medical concepts in UMLS. Thus, our mapping strategy that maps Chinese medical entities to the UMLS can provide the possibility of building Chinese medical ontologies inspired by UMLS.

Furthermore, the UMLS does not have sufficient coverage for other languages, such as French, Spanish, Hebrew, Chinese, and Japanese. With the help of web-based translation engines, our approach provides the ability to map cross-lingual medical entities to the UMLS. By applying different translation tools, our approach can be widely applied to many languages for mapping medical entities to the UMLS. This will enable more cross-lingual medical entities to be mapped to the UMLS, increase the multilingual coverage of the UMLS, and upgrade the UMLS to a comprehensive medical unification system globally. The enhancement of multilingual coverage for the UMLS will also facilitate a variety of the UMLS-based medical informatics processing tools, such as MetaMap, to be used in other languages.

### Limitation

A substantial limitation in this study is memory usage, as we decide to deploy our approach as a service. Since we map each UMLS term to a 768-dimensional semantic word vector and a 35,603-dimensional TF-IDF word vector to represent the semantic and string features of the term, 56-GB RAMs (Random Access Memory) are required when we represent all UMLS entities with disordered semantic types in semantic and string vector spaces. If we represent all UMLS terms in future research, more RAMs are necessary. Such a large requirement of RAMs heavily restricts the deployment of our approach and slows down the computation speed. Therefore, in future research, we will explore a hierarchical deployment architecture that implements dynamic concepts and term representations by referring to the hierarchy structure of the UMLS with the aim of considerably reducing memory usage.

The semantic types of the evaluation datasets belong to the “Disorder” semantic group. Terms with other semantic types are not tested in this study. The reason for this is the lack of “Golden Standard” datasets for the evaluation. There is no well-constructed dataset to evaluate the performance of our approaches on other semantic types. Therefore, in future work, we will further improve the RealWorld dataset to evaluate the mapping performance of our strategies on entities with other semantic types. Another limitation on evaluation datasets is the lack of lexical variants and synonyms of Chinese medical terms. The abundance of synonyms in evaluation datasets will affect the difficulty of the evaluation task. We will systematically investigate the influence of lexical variants and synonyms in the evaluation process.

Furthermore, inherently, the linear combination method proposed in this study only optimizes parameters *α* and *β*. Therefore, only similarities of string and semantic contribute to the final mapping score. Intrinsic knowledge joining of strings and semantics is considered inadequate. We need to further explore an efficient way to systematically learn string and semantic representation knowledge during training process. A possible solution with knowledge infusing for this kind of goal is to design a suitable learning scheme and a loss function that consider both string and semantic meanings during the PLM training process. Our main goal for future research is to develop the type of language model that fully integrates knowledge of medical concepts at the string and semantic levels. This type of language model not only facilitates the mapping efficacy but also reduces the complexity of model deployment and application.

### Conclusion

In this paper, we present an effective strategy, the linear combination method, based on SapBERT and TF-IDF, for mapping Chinese medical entities to the UMLS with the help of multiple translation engines. The linear-combination mapping method presented in this study overcomes the obstacle of inadequately developed Chinese medical terminology systems. By mapping Chinese medical entities to the UMLS, our research enables Chinese medical entities to be well-organized like UMLS, which provides a feasible technical solution for Chinese ontology construction. Furthermore, our method can also be widely used in downstream tasks to automatically map Chinese medical terms to standard UMLS concepts, thereby facilitating fine-grained medical knowledge representation and other advanced intelligent medical applications.

## Data Availability

The datasets used in this study are publicly available from the following sources: UMLS (https://uts.nlm.nih.gov/uts/umls/home), CHPO (https://www.chpo.net), ICD10-CN (http://www.nhc.gov.cn/cms-search/xxgk/getManuscriptXxgk.htm?id=52905), and the RealWorld (https://www.jianglab.tech/PhenoSSU/tool). We published our code on GitHub about how to generate training/evaluation sets from these datasets. Note that to make use of our code and data, one must first obtain access to the UMLS and CHPO.

## SUPPLEMENTARY MATERIALS

Supplementary 1 Sections S1 to S6. Figs. S1 to S3. Tables S1 to S5.
