# Supplementary material for: Mapping Chinese Medical Entities to the Unified Medical Language System
Source: Health Data Sci. 2023 Mar 30;3:0011. doi: 10.34133/hds.0011 (PMC10880171; doi:10.34133/hds.0011)
Supplement: Supplementary 1 — Sections S1 to S6. Figs. S1 to S3. Tables S1 to S5. [file hds.0011.f1.docx]

Supplementary Materials

1. Translation Evaluation

In this study, we use three internet translation engines, Baidu Fanyi, Youdao and Tencent Translator, to translate Chinese medical terms into English and evaluate their performances on the Chinese-English Medical Vocabulary published by the National Health Commission of the People's Republic of China (NHC). The evaluation results are shown in Table S1. We compare the translation of Chinese medical terms in the NHC vocabulary with their English counterparts. As shown in Table S1, 32.09%, 25.28%, and 32.98% of the NHC vocabulary can be translated into accurate English counterparts using Baidu Fanyi, Youdao, and Tencent Translator, respectively. The combination of translation from three engines (later called multisource translation) improves the proportion of accurate translations to 38.77%.

Table S1. Quality evaluation for web-based translation engines and multiple unified translation

|  | Matched Coverage | BLEU-1 |
| --- | --- | --- |
| Baidu Fanyi | 32.09% | 0.63 |
| Youdao | 25.28% | 0.64 |
| Tencent Translator | 32.98% | 0.62 |
| **Multisource translation** | **38.77%** | **0.74** |

To comprehensively evaluate the translation quality, we applied the BLEU metric to the NHC vocabulary and its translations. In Table S1, the BLEU-1 scores of Baidu Fanyi, Youdao and Tencent Translator are 0.63, 0.64, and 0.62, respectively, which are above the current average level of Chinese English translation. The BLEU-1 score of multisource translation is 0.74, which shows that multisource translation improves the translation quality.

In addition, we observed that even if the translation of Chinese medical terms is different in writing from the English counterparts, it still has the same meaning. For example, “lithium salt poisoning” translated from “锂盐中毒” has the same meaning as “lithium intoxication”, the counterpart of the Chinese medical term in NHC. Based on the evaluation, it is practical to use a translation engine and multisource translation scheme to facilitate mapping between Chinese medical terms and the UMLS.

1. Evaluation Dataset

Chinese medical entities are not organized comprehensively due to the lack of well-developed terminology systems. There is no other Chinese medical term dataset (in disorder semantic group) that is mapped to the UMLS except ICD10-CN and CHPO. These two datasets are important vocabularies which facilitate the evaluation of mapping performance. Furthermore, to extend the representativeness of evaluation datasets, we constructed a dataset called RealWorld in this study. Terms in the RealWorld datasets were collected from real-world medical corpus. Terms in the RealWorld dataset were manually mapped to the UMLS and reviewed by medical students and professionals. Details about evaluation datasets were described as follows.

1. ICD10-CN. The ICD-10 Chinese version was created by the National Health Committee (NHC) in China and contains 11451 disease terms. All of these medical terms were included in the ICD10-CN dataset. Terms in the dataset were mapped to UMLS through the ICD10-AM codex defined in UMLS Metathesauruses.

2. CHPO. The CHPO ontology was created by the CHPO organization, which focuses on Chinese Human Phenotype Ontology (CHPO) development and management. All 13,655 terms in the CHPO thesaurus were collected into the evaluation dataset and mapped to UMLS according to the HPO codex in UMLS.

3. RealWorld dataset. Medical terms are official, and the full names used in the ICD10-CN and CHPO datasets. However, in real world applications, abbreviations or common names of medical terms are often used. For example, in EHRs or internet medical health documents, “upper respiratory tract infection” (“上呼吸道感染”) may be written as “上感”. To compensate for the insufficient real world medical terms in the ICD10-CN and CHPO, the RealWorld dataset was constructed using terms from real world medical documents such as clinical EHRs, online health communities, case reports or others.

Y.Q. and L.C. manually mapped these medical entities to UMLS. L.D. reviewed the mapping results. A total of 2824 medical terms were mapped to UMLS by Y.Q. and L.C. independently. There are 226 different mappings in the mapping results, and the Inter-Annotator Agreement (IAA) is $\kappa=0.92$. After confirmation of the different mapping results, L.D. reviewed all the mapping results. IAA is defined as follows:

$$\kappa=\frac{P_{0}-P_{e}}{1-P_{e}}$$

where $\kappa$ is Inter-Annotator Agreement ranging from 0 to 1. Higher $\kappa$ values represent better agreement. $P_{0}$ is the actual agreement, which refers to the ratio of identical annotations between annotators. $P_{e}$ is the expected agreement that refers to the expected ratio of identical annotations between annotators. Through manual linking, review, and consistency checks, we believe that the RealWorld dataset constructed in this study has been comprehensively mapped to UMLS, which can be used to objectively evaluate the mapping performance of various methods for mapping Chinese medical entities to UMLS.

To explore the representativeness of the RealWorld dataset, we made statistics on the semantic types distribution of medical terms in the RealWorld dataset. Table S2 shows that all semantic types in “Disorder” semantic group are included in the RealWorld dataset. We also observed that the distribution of semantic types in the RealWorld dataset is similar to the “Disorder” semantic group. The main semantic types in “Disorder” semantic group like T047-Disease or Syndrome, T184-Sign or Symptom, and T033-Finding are also the key components in the RealWorld dataset.

Table S2. Number of terms in the RealWorld dataset which tagged with Top10 semantic types in “Disorder” semantic group

| Semantic Type | No. of Terms in RealWorld |
| --- | --- |
| T033 - Finding | 410 |
| T037 - Injury or Poisoning | 131 |
| T047 - Disease or Syndrome | 1296 |
| T191 - Neoplastic Process | 301 |
| T046 - Pathologic Function | 132 |
| T184 - Sign or Symptom | 338 |
| T019 - Congenital Abnormality | 73 |
| T048 - Mental or Behavioral Dysfunction | 63 |
| T190 - Anatomical Abnormality | 36 |
| T020 - Acquired Abnormality | 28 |

In conclusion, according to component exploration, the RealWorld dataset represents the distribution of “Disorder” semantic group adequately.

At last, Table S3 lists some terms and their UMLS CUI code included in the three evaluation datasets.

Table S3. Examples of Evaluation Datasets

| ICD10-CN | CHPO | RealWorld |
| --- | --- | --- |
| C0008354: 霍乱，未特指的 (Cholera, unspecified) | C3164374: 肺动脉瓣异常 (Abnormality of the pulmonary valve) | C0041912: 上感 (Upper respiratory tract infection) |
| C0152502: 阿米巴肺脓肿 (Amoebic lung abscess) | C0238462: 甲状腺髓样癌 (Medullary thyroid carcinoma) | C0018965: 尿血 (Hematuria) |
| C1260915: 潜伏性晚期梅毒 (Late syphilis, latent) | C4025670: 染色体分离异常 (Abnormality of chromosome segregation) | C0332601: 满月脸 (Cushingoid facies) |
| C0162316: 未特指的缺铁性贫血 (Iron deficiency anaemia, unspecified) | C1839533: 高谷氨酰胺血症 (Hyperglutaminemia) | C0001973: 嗜酒 (alcoholism) |
| …… | …… | …… |

Table S4. Overlaps between evaluation datasets

| Overlap | ICD10-CN  (11451 in total) | CHPO  (13665 in total) | RealWorld  (2824 in total) |
| --- | --- | --- | --- |
| ICD10-CN | - | - | - |
| CHPO | 366 (674) | - | - |
| RealWorld | 10 (509) | 80 (380) | - |

*Note:* numbers out of brackets represent the overlaps at term level. Numbers in brackets represent overlaps at concept level.

1. String-based Strategy

The string-based strategy contains three methods: MetaMap, ElasticSearch and the TF-IDF BoW model. For these methods, the similarity scoring functions between queries and candidate concepts are all based on the information from strings. MetaMap scores the similarity based on string features, which include centrality, variation, coverage and cohesiveness. MetaMap focuses on string level consistency. ElasticSearch uses the BM25 algorithm, which focuses on the word or token relevance between the query and the candidate. Elasticsearch indexes the candidate concepts and recommends the most similar candidates based on the BM25 score between the query and indices. If there are more important mutual tokens between the query and the candidate, the BM25 score will be higher. The TF-IDF BoW model focuses on the character-level n-gram relevance between the query and the candidate. In the study, entities in the UMLS2020AB disorder semantic group are used as the corpus to fit the TF-IDF BoW model. Then, translated Chinese medical terms and concept candidates are transformed into sparse vectors by this model. Based on the cosine similarity, the most similar candidate concepts can be recommended.

- 1. MetaMap

MetaMap is a program that can recognize and normalize the medical entities of the UMLS in documents developed by the NIH. It consists of three parts, including Specialist lexical tools, UMLS Metathesauruses and Evalution Fucntions. The mapping process of MetaMap is divided into five steps:

1. Parse the text into noun phrases
2. Generate the variants for the noun phrase
3. Form the candidate set of all Metathesaurus strings containing one of the variants
4. For each candidate, compute the mapping from the noun phrase and calculate the strength of the mapping using an evaluation function
5. Combine candidates involved with disjoint parts of the noun phrase, recompute the match strength based on the combined candidates and select the best mapping according to the evaluation score

In the study, Metamap is used with the UMLS2020AB metathesauruses as a mapping tool that links translated Chinese medical terms to the CUI. In the practice of MetaMap, option -Z is set for short fragment normalization, and option J is set as “-J acab, anab, bact, cgab, comd, dsyn, emod, fndg, inpo, mobd, patf, sosy” to restrict the candidate concepts in the UMLS disorder semantic group.

- 1. Elasticsearch

Elasticsearch is a search engine based on the BM25 algorithm. BM25 is a ranking function that scores each index according to its relevance to the query. Similar to TF-IDF, term frequency (TF) and inverse document frequency (IDF) are computed for each token in a query. The relevance score is summed by TF for each token, which is punished by IDF for a less important token. On this basis, the BM25 algorithm adjusts the parameters of TF and IDF to avoid the impact of unsaturated TF and long documents on the relevance score. The BM25 ranking function is as follows:

$$score\left( T,Q \right)=\sum_{i=1}^{n} \text{IDF}\left( t_{i} \right)\cdot\frac{f\left( t_{i},T \right)\cdot\left( k_{1}+1 \right)}{f\left( t_{i},T \right)+k_{1}\cdot\left( 1-b+b\cdot\frac{\left| Q \right|}{\text{avgdl}} \right)}$$

Q is the query, T is the candidate index, N is the size of the search index, n is the document frequency, and t is the token. k and b are fitting parameters. In the study, the default parameters of Okapi BM25 are used, which are k = 1.2 and b = 0.75. In the practice of Elasticsearch, entities with CUI codes in the UMLS2020AB disorder semantic group are imported by Logstash to the Elasticsearch engine, and then they are indexed. Translated Chinese medical terms are searched in the index. Then, candidate concepts are retrieved based on the ranking of the BM25 scores.

- 1. Bag-of-words with TF-IDF


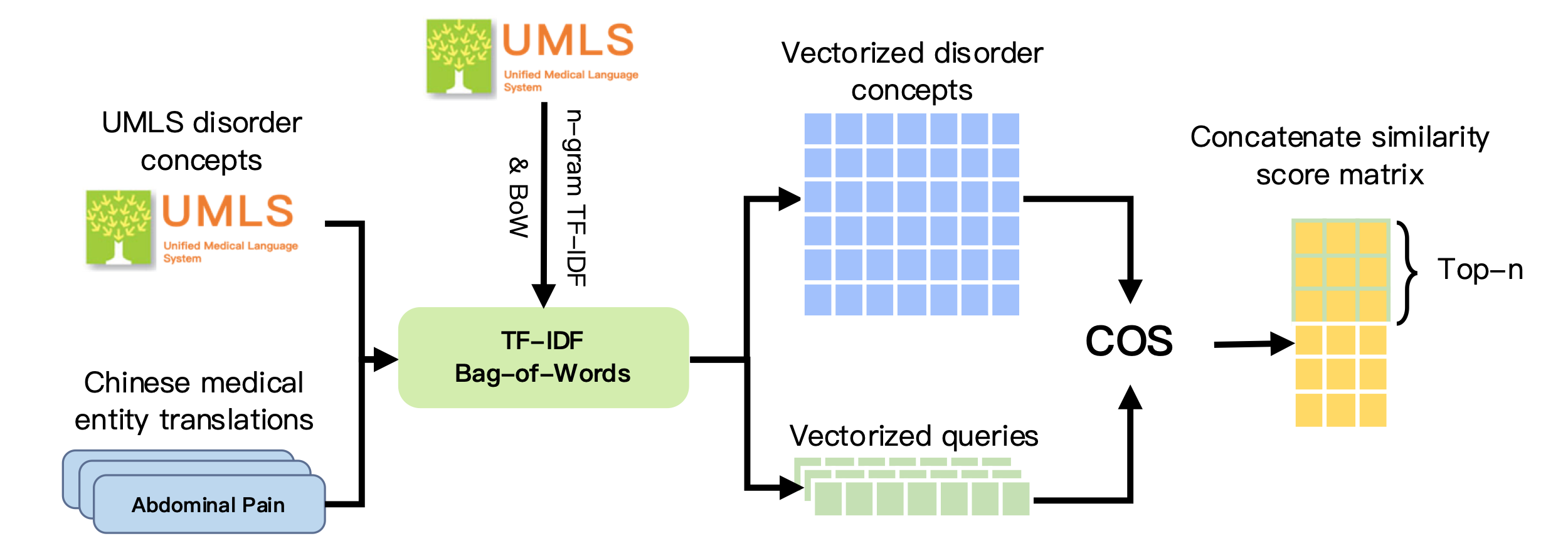


Figure S1. Mapping workflow of the TF-IDF BoW method

Before neural language models were developed, the bag-of-words (BoW) model was the most common statistical language model. The TF-IDF character-level n-gram bag-of-words model statistically calculates each n-gram sub-word’s TF-IDF score in the corpus and can be used to create feature vectors with TF-IDF scores. For example, when n = 2, “diabetes” can be represented by a sparser vector whose entries equal the n-grams’ (d, i, a, b, e, t, s, di, ia, ab, be, et, te, es) TF-IDF score in the corpus. The TF-IDF score of n-grams is calculated as follows:

$tf\left( st,d \right)=\frac{f_{st,d}}{\sum_{t'\in d} f_{t',d}}$

where $f_{st,d}$ is the raw count of an n-gram in a document, $\sum_{t'\in d} f_{t',d}$ is the total number of n-grams, and $tf\left( st,d \right)$represents the frequency of the term.

$idf\left( st,D \right)=log\frac{N}{\left| \{d\in D:st\in d\} \right|}$

$N$ is the total number of documents in the corpus. $\left| \{d\in D:st\in d\} \right|$ represents the number of documents where the term $st$ appears. $idf\left( st,D \right)$ represents the inverse document frequency of the term in the corpus. Finally, TF-IDF is calculated as follows:

$\mathrm{tfidf}\left( st,d,D \right)=tf\left( st,d \right)\cdot idf\left( st,D \right)$

In the study, entities in the UMLS2020AB disorder semantic group are used as the corpus to fit the TF-IDF BoW model. Then, translated Chinese medical terms and concept candidates are transformed into sparse vectors by this model, where the vector length equals the n-gram dictionary length. Each entry in a sparse vector equals an n-gram TF-IDF value in the corpus if that n-gram occurs in the term being transformed or 0 if it does not. Based on the cosine similarity score between sparse vectors, the most similar candidate concepts can be recommended. This process is shown in Figure S1.

1. Semantic-based Strategy

For the semantic-based strategy, UMLS entities are represented by pretrained language models (PLMs). Each UMLS entity is embedded as a 768 vector from the last hidden state of the $CLS$ token in PLMs. In the study, entities in the UMLS2020AB disorder semantic group are used as candidate concepts and embedded by PLMs as a matrix. Dense vectors of entities were computed as follows:

$e^{d_{LM}}=\mathbf{LM}\left( t \right)\left[ CLS \right]$

where $e^{d}$ represents the semantic vector of the query and candidate encoded by the language model. $\mathbf{LM}$ denotes the language model. $CLS$ is a special token generated by the language model, which usually stands for the last layer output of the language model.

In the mapping stage, Chinese medical entities are translated into English and embedded by PLMs, such as candidates. By calculating and ranking the cosine similarity scores between embeddings of each Chinese medical entity and candidate concepts, Top-N candidate concepts can be retrieved. The cosine similarity score is computed as follows:

$S_{\mathrm{semantic}}\left( q, c \right)=\cos\left( \theta\right)=\frac{e_{q}^{d} \cdot e_{c}^{d}}{|e_{q}^{d} ||e_{c}^{d} |}$

The process of the semantic-based strategy is shown in Figure S2.


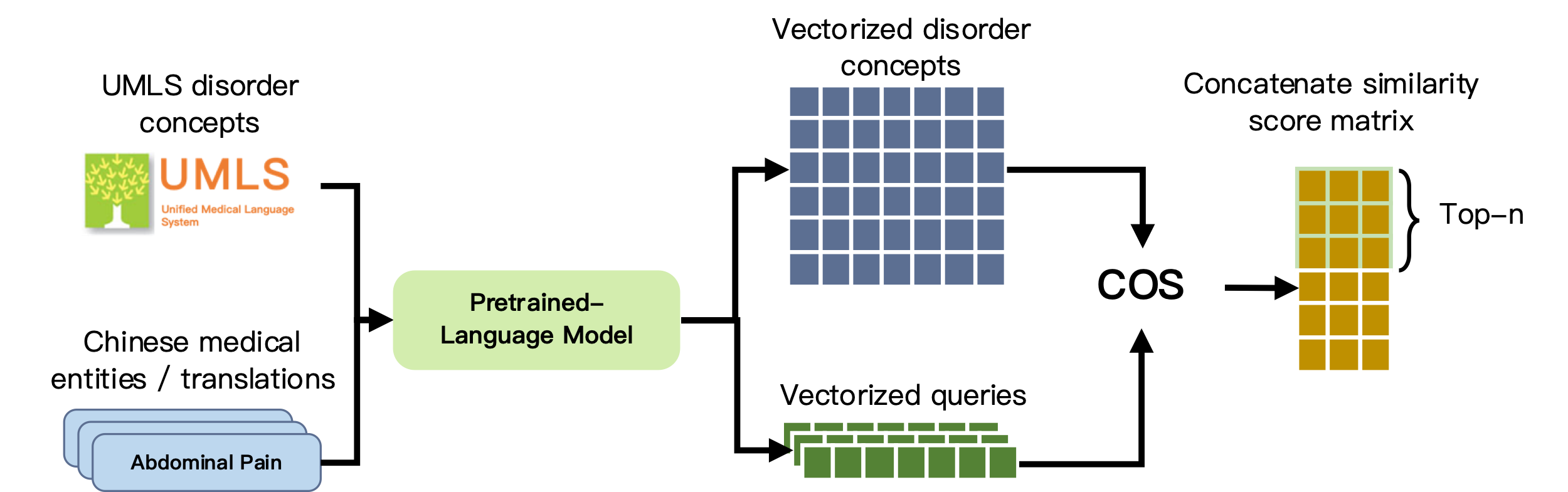


Figure S2. Mapping workflow of semantic strategy

1. Performance Difference Test

To test the significance of mapping performance differences between models, we performed McNemar’s test for linear-combination method and others. The results are shown in the Table S5. According to the test results, there is a significant difference between linear-combination method and others. Thus, linear combination method significantly outperforms other mapping methods we explored in this study.

Table S5. McNemar’s test p-value between linear-combination method (SapBERT + TF-IDF BoW) and other methods (NaN in the table means that p-values are too close to 0 to show.)

| Linear-combination | ICD10-CN | | | CHPO | | | RealWorld | | |
| --- | --- | --- | --- | --- | --- | --- | --- | --- | --- |
|  | @1 | @5 | @10 | @1 | @5 | @10 | @1 | @5 | @10 |
| MetaMap | NaN | NaN | NaN | NaN | NaN | NaN | 1.50×10^-105^ | 6.86×10^-140^ | 5.25×10^-151^ |
| Elasticsearch | NaN | NaN | NaN | NaN | NaN | NaN | 1.78×10^-60^ | 3.90×10^-74^ | 3.38×10^-74^ |
| n-gram TF-IDF BoW (n=2) | NaN | 7.82×10^-249^ | 1.67×10^-210^ | NaN | NaN | NaN | 1.39×10^-35^ | 7.55×10^-44^ | 1.77×10^-43^ |
| n-gram TF-IDF Bow (n=3) | NaN | 1.16×10^-231^ | 1.38×10^-191^ | NaN | NaN | NaN | 4.78×10^-43^ | 6.27×10^-38^ | 3.27×10^-40^ |
| BERT | NaN | NaN | NaN | NaN | NaN | NaN | 4.81×10^-72^ | 2.76×10^-95^ | 1.92×10^-104^ |
| BioBERT | NaN | NaN | NaN | NaN | NaN | NaN | 5.20×10^-50^ | 1.49×10^-59^ | 6.59×10^-61^ |
| SapBERT | 1.02×10^-159^ | 1.01×10^-88^ | 1.69×10^-78^ | 1.08×10^-166^ | 9.37×10^-161^ | 6.08×10^-162^ | 2.66×10^-06^ | 4.79×10^-06^ | 0.00365 |
| *SapBERT + TF-IDF BoW* |  |  |  |  |  |  |  |  |  |
| z-score | 1.15×10^-106^ | 2.71×10^-58^ | 8.29×10^-42^ | 4.89×10^-191^ | 3.33×10^-146^ | 1.22×10^-142^ | 1.64×10^-18^ | 1.07×10^-18^ | 3.30×10^-17^ |
| Min-Max | 5.51×10^-84^ | 4.67×10^-45^ | 1.48×10^-35^ | 2.89×10^-140^ | 1.39×10^-118^ | 1.46×10^-110^ | 4.93×10^-11^ | 7.56×10^-12^ | 1.35×10^-10^ |
| Tanh | 3.60×10^-105^ | 8.57×10^-58^ | 1.47×10^-41^ | 2.14×10^-185^ | 6.59×10^-146^ | 6.15×10^-143^ | 5.85×10^-17^ | 3.92×10^-19^ | 1.59×10^-16^ |

1. Linear Combination Optimization and Significant Test

We randomly select N entities from the UMLS2020AB disorder semantic group and translated them to Chinese as a simulation dataset. Cosine similarity scores between the dataset and candidate concepts are computed by the TF-IDF-BoW model and SapBERT PLM. We then combine the SapBERT semantic similarity score and the TF-IDF-BoW string similarity score as the integration score with parameters $\alpha$ and $\beta$.

$S_{integration}=\alpha S_{semantic}+\beta S_{string}$

By optimizing $\alpha$ and $\beta$, the integration score has the potential to improve the performance of mapping Chinese medical entities to UMLS. We optimized $\alpha$ and $\beta$ by maximizing the marginal probability of positive candidate concept synonyms in Top N recommendations, where N = 20 in this study. The top 20 recommendations are selected from the disorder semantic group dictionary according to the integration scores. The marginal probability of the synonym in recommendations is as follows:

$P\left( n_{i} | q \right)=\frac{\exp\left( S_{\mathrm{integrate}}\left( n_{i},q \right) \right)}{\sum_{i=1..N} e\mathrm{xp}\left( S_{\mathrm{integrate}}\left( n_{i},q \right) \right)}$

where q stands for the query and n is the recommendation. Then, the marginal probability of all positive synonyms in recommendations is defined as follows:

$P^{'}\left( q ,N \right)=\sum_{n\in N;EQUAL\left( q, n \right)=1} P\left( n_{i}|q \right)$

where $EQUAL\left( q ,n \right)=1$ denotes entities in recommendations that are the synonyms for the query that belong to the same concept. Our goal is maximizing the marginal probability of a positive candidate concept synonyms in the Top N recommendations. Thus, the loss function is defined as follows:

$Loss=-\frac{1}{Q}\sum_{i=1}^{Q} \log P^{'}\left( q_{s_{i}},N_{q_{s_{i}}} \right)$

where Q is the size of the simulation dataset.

To ensure that the number of samples in the simulation dataset for parameter optimization is sufficient, we randomly select Q (Q = {100, 500, 1000, 2500, 5000, 10000, 15000, 20000}) entities from the disorder semantic group in the UMLS and translate them to Chinese as the simulation dataset. For each Q, we repeat random selection and training 30 times. The optimized $\alpha$, $\beta$ and$\alpha/\beta$ of each repetition are shown in Figure S3.


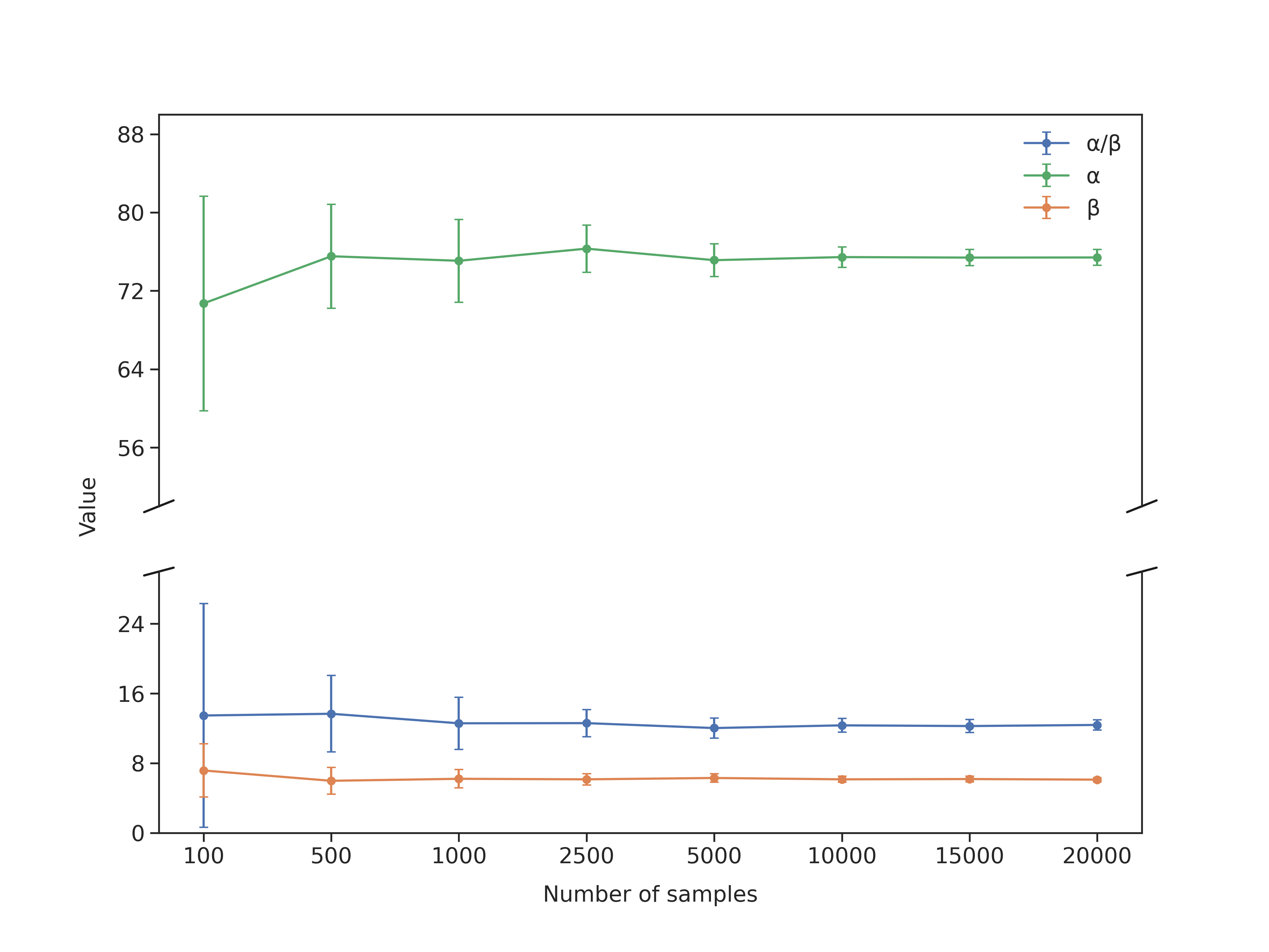


Figure S3. Parameter optimization and statistical results.

As the number of samples in the simulation dataset increases, α and β are stabilized at a consistent level during each random selection and training process. We run Bartlett’s test and ANOVA to determine the minimum Q required for training. This shows that when Q is up to 10000, there is no significant change in the mean and variance of $\alpha, \beta and \alpha/\beta$. The result of Bartlett’s test for Q = 10000, 15000 and 20000 is p = 0.30, and the result of ANOVA for Q = 10000, 15000 and 20000 is p = 0.80.
